# Functional Analysis of Mating Type Genes and Transcriptome Analysis during Fruiting Body Development of *Botrytis cinerea*

**DOI:** 10.1128/mBio.01939-17

**Published:** 2018-02-13

**Authors:** Sander Y. A. Rodenburg, Razak B. Terhem, Javier Veloso, Joost H. M. Stassen, Jan A. L. van Kan

**Affiliations:** a Laboratory of Phytopathology, Wageningen University, Wageningen, The Netherlands; b Wageningen University, Bioinformatics Group, Wageningen, The Netherlands; c Department of Plant Physiology, Faculty of Sciences, University of A Coruña, A Coruña, Spain; Cornell University

**Keywords:** ascospore, epigenetic regulation, plant disease, sexual reproduction, transcriptome

## Abstract

*Botrytis cinerea* is a plant-pathogenic fungus producing apothecia as sexual fruiting bodies. To study the function of mating type (*MAT*) genes, single-gene deletion mutants were generated in both genes of the *MAT1-1* locus and both genes of the *MAT1-2* locus. Deletion mutants in two *MAT* genes were entirely sterile, while mutants in the other two *MAT* genes were able to develop stipes but never formed an apothecial disk. Little was known about the reprogramming of gene expression during apothecium development. We analyzed transcriptomes of sclerotia, three stages of apothecium development (primordia, stipes, and apothecial disks), and ascospores by RNA sequencing. Ten secondary metabolite gene clusters were upregulated at the onset of sexual development and downregulated in ascospores released from apothecia. Notably, more than 3,900 genes were differentially expressed in ascospores compared to mature apothecial disks. Among the genes that were upregulated in ascospores were numerous genes encoding virulence factors, which reveals that ascospores are transcriptionally primed for infection prior to their arrival on a host plant. Strikingly, the massive transcriptional changes at the initiation and completion of the sexual cycle often affected clusters of genes, rather than randomly dispersed genes. Thirty-five clusters of genes were jointly upregulated during the onset of sexual reproduction, while 99 clusters of genes (comprising >900 genes) were jointly downregulated in ascospores. These transcriptional changes coincided with changes in expression of genes encoding enzymes participating in chromatin organization, hinting at the occurrence of massive epigenetic regulation of gene expression during sexual reproduction.

## IMPORTANCE

Fungal fruiting bodies are formed by sexual reproduction. We studied the development of fruiting bodies (“apothecia”) of the ubiquitous plant-pathogenic ascomycete *Botrytis cinerea*. The role of mating type genes in apothecium development was investigated by targeted mutation. Two genes are essential for the initiation of sexual development; mutants in these genes are sterile. Two other genes were not essential for development of stipes; however, they were essential for stipes to develop a disk and produce sexual ascospores. We examined gene expression profiles during apothecium development, as well as in ascospores sampled from apothecia. We provide the first study ever of the transcriptome of pure ascospores in a filamentous fungus. The expression of numerous genes involved in plant infection was induced in the ascospores, implying that ascospores are developmentally primed for infection before their release from apothecia.

## INTRODUCTION

Sexual reproduction permits organisms to generate new combinations of alleles and thereby offers the possibility of increasing the fitness of (part of) their offspring. Sexual spores of fungi can survive under adverse conditions (1, 2), but their main biological function is in dispersal. Sexual spores of plant-pathogenic fungi can be dispersed by rain or wind, sometimes over long distances, and can subsequently serve as inoculum for new infections (3–6). In ascomycete fungi, sexual compatibility is determined by two opposite mating type (*MAT*) loci, designated *MAT1-1* and *MAT1-2* (7–9). Ascomycetes can have either heterothallic or homothallic sexual reproduction systems. In a heterothallic species, mating occurs between two isolates with opposite mating types. The opposite *MAT* loci of ascomycetes are generally present at the same chromosomal location and therefore genetically allelic; however, they lack sequence similarity and are often referred to as “idiomorphs” rather than alleles (10). Each idiomorph contains at least one gene encoding a transcription factor. By convention, the *MAT1-1* idiomorph encodes a protein with an α domain, while *MAT1-2* encodes a protein with a high-mobility-group box (HMG box) (9, 11). Homothallic species are capable of self-fertilization, as they carry both core *MAT* genes (*MAT1-1-1* and *MAT1-2-1*) in one genome (12–15), usually next to each other, although the model fungus *Aspergillus nidulans* serves as an exception since its core *MAT* genes are located on separate chromosomes (16).

*Botrytis cinerea* is a heterothallic ascomycete fungus in the class *Leotiomycetes*, order *Helotiales*, family *Sclerotiniaceae*. A gapless community-annotated genome sequence is available (17). As a member of the *Helotiales*, *B. cinerea* develops a fruiting body called an apothecium (18, 19), consisting of an exposed hymenium on top of a stipe. Apothecia are carpogenic from a sclerotium, a melanized survival structure that contains a reservoir of nutrients to support apothecium development (20) and usually resides in plant residues in the topsoil layer. By their emergence from sclerotia and their phototropic growth (21), apothecia of *Sclerotiniaceae* function as an elevated launch platform that facilitates the discharge of ascospores (22), which act as primary inoculum for plant infection, especially in early spring when the asexual conidia are scarce.

The *B. cinerea MAT1-1* locus contains two genes: *MAT1-1-1*, encoding the α domain protein, and *MAT1-1-5*, of unknown function. The *MAT1-2* locus also contains two genes: *MAT-1-2-1*, encoding the HMG box protein, and a second gene of unknown function. This *B. cinerea* gene was initially named *MAT1-2-4* (15) and has an ortholog in *Sclerotinia sclerotiorum*; however, an ongoing revision of ascomycete *MAT* genes resulted in their being renamed *MAT1-2-10* (9). For this reason, the new name will be adopted for these *B. cinerea* and *S. sclerotiorum* genes from here onward. Both *MAT* loci in *B. cinerea* are flanked by the *APN2* gene (ortholog of the *Saccharomyces cerevisae* gene encoding a DNA lyase) and the *SLA2* gene (ortholog of the *S. cerevisiae* gene encoding a cytoskeletal protein), which are convergently transcribed toward the *MAT* loci (15, 23). Except for several *Dothideomycetes*, most filamentous ascomycetes with heterothallic reproductive lifestyles possess *MAT* loci that are also surrounded by *APN2* and *SLA2* genes, suggesting that this represents the ancestral configuration in ascomycete fungi (7, 9). *S. sclerotiorum*, a close relative of *B. cinerea*, has a homothallic reproductive mode and carries four linked *MAT* genes (*MAT1-1-1*, *MAT1-1-5*, *MAT1-2-1*, and *MAT1-2-10*), which are equivalent to the heterothallic *MAT1-1* and *MAT1-2* loci in *B. cinerea* (15). Similarly, the homothallic species *Sordaria macrospora* carries four linked *MAT* genes (*MAT1-1-1*, *MAT1-1-2*, *MAT1-1-3*, and *MAT1-2-1*), equivalent to the genes in both idiomorphs of its heterothallic relative *Neurospora crassa* (24, 25).

Several studies have focused on unraveling the functions of mating type genes in ascomycetes by targeted mutagenesis, primarily in model fungi (26–31). Doughan and Rollins (32) recently described a functional analysis of genes in the *MAT* locus of *S. sclerotiorum* and reported that mutants in the *MAT1-1-1*, *MAT1-1-5*, and *MAT1-2-1* genes were entirely sterile, while mutants in the *MAT1-2-10* gene were delayed in carpogenic germination and formed apothecia with aberrant morphology. So far, the functions of the four genes in the *B. cinerea MAT* locus in apothecium development have not been studied. The genome sequence and gene annotations of *B. cinerea* (15, 17) enabled the molecular dissection of apothecium development in *B. cinerea*, at both the transcriptional and the functional level.

This study aimed to identify the functions of *B. cinerea MAT* genes in apothecium development by targeted deletion and to perform a genome-wide transcriptome analysis during sexual reproduction using RNA sequencing (RNA-seq), which currently is the preferred method because of its sensitivity and quantitative accuracy (33–35) as well as its affordability. Expression profiling by RNA-seq was reported in numerous filamentous fungi (e.g., references 36 to 40); however, RNA-seq analyses in the context of sexual development are less abundant (41–45). Expression profiles observed during *B. cinerea* fruiting body development revealed concerted upregulation in the sexual ascospores of numerous genes, many of which are involved in interactions with plants, suggesting developmental priming for host invasion during sexual reproduction. Hints that epigenetic changes play a role in this regulation were obtained.

## RESULTS AND DISCUSSION

### Development of *Botrytis cinerea* apothecia.

**FIG 1:**
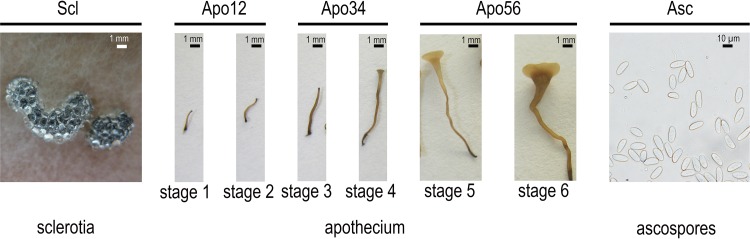
Different stages of sexual reproduction in *Botrytis cinerea*. Following fertilization of asexual resting structures (sclerotia), apothecium development is divided into six stages: primordia emerging (stage 1), primordia extending (stage 2), extended stipes before tip swelling (stage 3), stipes with swollen tips (stage 4), immature apothecium (stage 5), and mature apothecium with asci and ascospores (stage 6). Pure ascospores sampled from mature apothecial disks are shown in the right-hand image. The five samples used for transcriptome analyses are indicated at the top.

Fertilization of vernalized sclerotia of *B. cinerea* isolate SAS405 (*MAT1-2*) with microconidia of *B. cinerea* isolate SAS56 (*MAT1-1*), followed by incubation under appropriate conditions (18, 19), resulted in the formation of apothecia with asci and ascospores. Crosses were effective reciprocally, i.e., regardless of which isolate was used as the maternal parent (sclerotia) or the paternal parent (microconidia). Sexual structures began to emerge from sclerotia at 20 to 30 days postfertilization (dpf) and reached maturity at 30 to 60 days postemergence. Apothecial development was divided into six stages (Fig. 1): primordia emerging from sclerotium (stage 1, 20 to 30 dpf); primordia extending into stipes (stage 2, 24 to 35 dpf); fully extended stipes before onset of tip swelling (stage 3, 30 to 45 dpf); stipes with swollen tips before apothecial disk expansion (stage 4, 35 to 60 dpf); immature apothecial disk with a diameter of <3 mm and a pale color (stage 5, 40 to 70 dpf); and mature disk with a diameter of >5 mm and a light brown color and filled with asci containing eight ascospores (stage 6, 50 to 90 dpf).

### Functional analysis of the *MAT* genes by targeted deletion.

**FIG 2:**
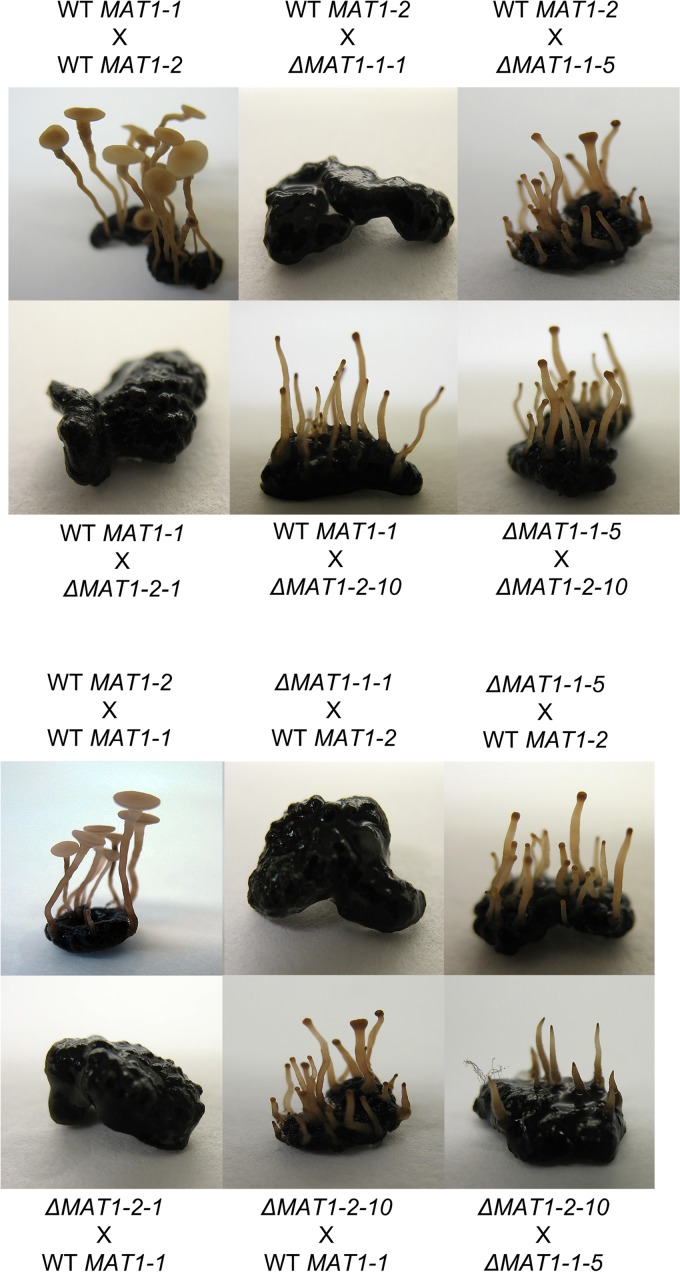
Results of crosses with *B. cinerea* mutants in *MAT* genes. The maternal parent (sclerotia) is mentioned first, and the paternal parent (microconidia) is second. The six images below the dashed line show the results for reciprocal crosses of the six images above. For each mutated target gene, results of one mutant are shown; identical results were obtained with two additional, independent deletion mutants.

In order to study the function of mating type genes *MAT1-1-1*, *MAT1-1-5*, *MAT1-2-1*, and *MAT1-2-10* in apothecium development, deletion mutants were generated by replacing the coding region of each gene with a hygromycin resistance cassette in wild-type strains SAS56 and SAS405 (see Fig. S1 in the supplemental material). The *MAT1-1-5* gene was also deleted in the genetic background of strain B05.10. Between three and six independent deletion mutants were obtained for each of the four *MAT* genes. All phenotypic analyses were carried out on three independent deletion mutants for each gene, and results were identical for independent mutants. To analyze whether the mutants had additional ectopic integrations, the copy number of the hygromycin resistance cassette was determined by quantitative PCR (qPCR) on genomic DNA and normalized to the single-copy housekeeping gene *Bcrpl5*. The deletion mutants had a single copy of the *hph* gene with the exception of one *MAT1-2-10* mutant (out of three tested) which contained ~10 additional copies in an unknown location(s). Growth rates and morphology of asexual structures (mycelium, sclerotia, and conidia) of all *ΔMAT1-1-1*, *ΔMAT1-1-5*, *ΔMAT1-2-1*, and *ΔMAT1-2-10* deletion mutants (including the *ΔMAT1-2-10* mutant with additional copies of the *hph* gene) were indistinguishable from those of the corresponding wild type. Reciprocal crosses were set up using two wild-type strains, SAS56 and SAS405, and four single-gene mutant strains (*ΔMAT1-1-1*, *ΔMAT1-1-5*, *ΔMAT1-2-1*, or *ΔMAT1-2-10*) in all relevant combinations (Table 1). In the control cross of wild-type sclerotia (acting as maternal parent) fertilized with wild-type microconidia (as paternal parent), apothecia developed as described above. In contrast, apothecia never developed and there was no sign of outgrowth of primordia when crosses were performed between wild-type strain SAS405 and the *ΔMAT1-1-1* mutant or between wild-type strain SAS56 and the *ΔMAT1-2-1* mutant (Fig. 2). Failure to develop sexual structures was also observed in reciprocal crosses, indicating that the *MAT1-1-1* and *MAT1-2-1* genes are essential for the initiation of sexual development, in both maternal and paternal tissues. Loss of fertility was also reported for deletion mutants in the *MAT1-1-1* or the *MAT1-2-1* gene of *S. sclerotiorum* (a close relative of *B. cinerea*), though ascogonia were normally formed in these mutants (32). We did not investigate the presence of ascogonia in these *B. cinerea* mutants, since it is unknown when and where the fertilization occurs in the sclerotia during the 4-week incubation period following fertilization.

10.1128/mBio.01939-17.1 FIG S1  Schematic representation of strategy for targeted deletion of *MAT* genes. Knockout of *BcMAT1-1-1*, *BcMAT1-1-5*, *BcMAT1-2-1*, and *BcMAT1-2-10* by targeted gene replacement. Organization of *BcMAT1-1-1*, *BcMAT1-1-5*, *BcMAT1-2-1*, and *BcMAT1-2-10* locus before and after homologous recombination. Orientations of the target gene and *HPH* are indicated by white and gray arrows, respectively. Upstream and downstream flanks of target genes are shown with open boxes. PCR analysis of wild-type strains SAS56 and SAS405 and knockout mutant strains. The genomic DNA of each strain was used to verify 5′ and 3′ homologous recombination and the absence of targeted genes in corresponding deletion mutants, respectively. Download FIG S1, PDF file, 0.3 MB. Copyright © 2018 Rodenburg et al. 2018 Rodenburg et al. This content is distributed under the terms of the Creative Commons Attribution 4.0 International license.

**TABLE 1:** Crosses performed between *B. cinerea* deletion mutants in *MAT* genes

Type of cross	Sclerotia (maternal parent)	Microconidia (paternal parent)	Primordia + stipes	Apothecial disks	Asci and ascospores
WT^d^ × WT	SAS56	SAS405	Yes	Yes	Yes
WT × WT	SAS405	SAS56	Yes	Yes	Yes
WT × mutant	SAS56	*ΔMAT1-2-1^a^*	No	No	No
WT × mutant	SAS56	*ΔMAT1-2-10^a^*	Yes	No	No
WT × mutant	SAS405	*ΔMAT1-1-1^b^*	No	No	No
WT × mutant	SAS405	*ΔMAT1-1-5^b^*	Yes	No	No
Mutant × WT	*ΔMAT1-2-1^a^*	SAS56	No	No	No
Mutant × WT	*ΔMAT1-2-10^a^*	SAS56	Yes	No	No
Mutant × WT	*ΔMAT1-1-1^b^*	SAS405	No	No	No
Mutant × WT	*ΔMAT1-1-5^b^*	SAS405	Yes	No	No
Mutant × mutant	*ΔMAT1-2-1^a^*	*ΔMAT1-1-1^b^*	No	No	No
Mutant × mutant	*ΔMAT1-2-1^a^*	*ΔMAT1-1-5^b^*	No	No	No
Mutant × mutant	*ΔMAT1-2-10^a^*	*ΔMAT1-1-1^b^*	No	No	No
Mutant × mutant	*ΔMAT1-2-10^a^*	*ΔMAT1-1-5^b^*	Yes	No	No
Mutant × mutant	*ΔMAT1-1-1^b^*	*ΔMAT1-2-1^a^*	No	No	No
Mutant × mutant	*ΔMAT1-1-1^b^*	*ΔMAT1-2-10^a^*	No	No	No
Mutant × mutant	*ΔMAT1-1-5^b^*	*ΔMAT1-2-1^a^*	No	No	No
Mutant × mutant	*ΔMAT1-1-5^b^*	*ΔMAT1-2-10^a^*	Yes	No	No
Control	*ΔMAT1-2-1^a^*	SMW^c^	No	No	No
Control	*ΔMAT1-2-10^a^*	SMW	No	No	No
Control	*ΔMAT1-1-1^b^*	SMW	No	No	No
Control	*ΔMAT1-1-5^b^*	SMW	No	No	No
Control	SAS56	SMW	No	No	No
Control	SAS405	SMW	No	No	No

a Mutant created in genetic background of SAS405.

b Mutant created in genetic background of SAS56.

c SMW, sterile Milli-Q water.

d WT, wild type.

**FIG 3:**
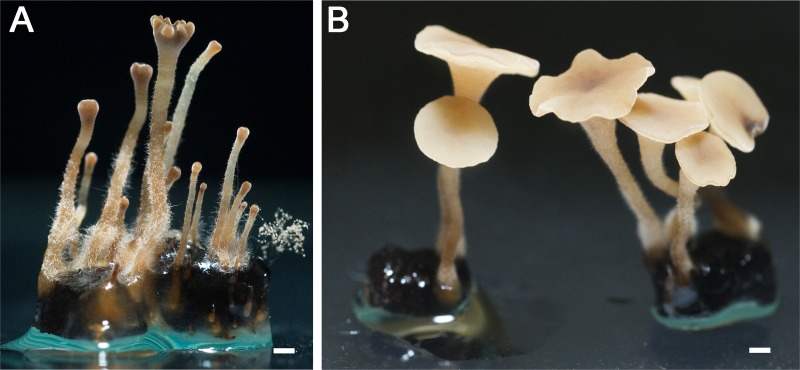
Closeup of defective stipes formed in a cross between wild-type sclerotia of SAS405 and a Δ*MAT1-1-5* mutant (A) and of a fully developed wild-type apothecium (B). The mutant stipe is blocked in transition to the apothecial disk and forms only a lobed, indented structure at the tip of the stipe. Crosses between wild-type sclerotia of SAS56 and *MAT1-2-10* deletion mutants, as well as between a *MAT1-1-5* deletion mutant and a *MAT1-2-10* deletion mutant, result in very similar phenotypes. White bar, 1 mm.

Sexual behavior of the *ΔMAT1-1-5* and *ΔMAT1-2-10* deletion mutants was distinct from that of *ΔMAT1-1-1* and *ΔMAT1-2-1* mutants (Fig. 2). Aberrant development of apothecia was observed in crosses between wild-type strain SAS405 and the *ΔMAT1-1-5* mutant, as well as in crosses between wild-type strain SAS56 and the *ΔMAT1-2-10* mutant. The emergence of primordia and development of stipes occurred similarly as in crosses between two wild-type strains; however, the stipes failed to develop into disks. Stipes did swell at the tip similarly to the wild type but failed to expand laterally. After several weeks of extended incubation, the stipes developed lobed, indented structures at the top (Fig. 3A), clearly distinct from wild-type apothecial disks (Fig. 3B). In these defective structures, obtained in crosses with *ΔMAT1-1-5* and *ΔMAT1-2-10* mutants, the development of croziers, asci, and ascospores was never observed. The failure of mutant stipes to make the developmental switch to apothecial disks was observed for three independent knockout mutants for either gene, and it occurred likewise in reciprocal crosses. Crosses between a *ΔMAT1-1-5* mutant and a *ΔMAT1-2-10* mutant yielded an identical defective phenotype as crosses between either single mutant and their corresponding wild-type mating partner (Fig. 2), suggesting that these two genes jointly control the transition from stipe to disk development.

The phenotype of these *B. cinerea* mutants differed from that of the corresponding mutants in *S. sclerotiorum*, in which the *ΔMAT1-1-5* mutant was entirely sterile and the *ΔMAT1-2-10* mutant showed severely delayed carpogenic germination and aberrant apothecium morphology (32). The transition from stipe to apothecial disk is typified by the formation of crozier cells in which karyogamy occurs (46, 47), and the resulting diploid nuclei subsequently enter meiosis to eventually form eight ascospores in an ascus. We propose that in *B. cinerea*, *MAT1-1-5* and *MAT1-2-10* act as (possibly dimeric) regulators that directly or indirectly control the formation of crozier cells and that the absence of either of these proteins results in failure to proceed to karyogamy, which blocks further downstream processes such as apothecial disk expansion. It remains to be demonstrated whether the MAT1-1-5 and MAT1-2-10 proteins indeed physically interact and how they impact on transcription and sexual development.

Attempts were made to complement the phenotype of *MAT* gene mutants. With the exception of the *MAT1-1-5* gene, complementation constructs in which wild-type genes, including flanking regulatory sequences, were cloned into vector pNR4 containing a nourseothricin resistance cassette (48) appeared unstable in *Escherichia coli*. Complementation could thus not be performed for the *ΔMAT1-1-1*, *ΔMAT1-2-1*, and *ΔMAT1-2-10* mutants. For the *MAT1-1-5* gene, the complementation construct was transformed into a *ΔMAT1-1-5* mutant. Three independent transformants with an ectopic insertion of the intact *MAT1-1-5* gene (including flanking sequences) in the *ΔMAT1-1-5* mutant background were tested in crosses, and all failed to show recovery of normal mating behavior. Ectopic integration of the intact *MAT1-1-5* gene may have resulted in an inadequate level or temporal pattern of transcription, either because of the wrong chromatin context or because of the occurrence of meiotic silencing by unpaired DNA (MSUD) in an ectopic location, as was reported for *Neurospora crassa* crosses using a mutant strain that contains the *matA* gene in an ectopic location (49). The *MAT1-1-5*-complemented transformants were not analyzed further.

### Sampling for analysis of gene expression during sexual development.

*B. cinerea* apothecia are an attractive resource for transcriptome analysis compared to other ascocarps such as cleistothecia, perithecia, and pseudothecia. Apothecia develop from sclerotia in an aqueous environment (18, 19), and the distinct developmental stages are in the millimeter-to-centimeter size range (Fig. 1). Hence, fairly pure tissue samples could easily be obtained. The fruiting body tissue samples were devoid of vegetative mycelium and sclerotial tissue. The use of laser microdissection to obtain ascocarp tissues, as performed in *Sordaria macrospora* (50), was thus not required for *B. cinerea*. Samples representing five different stages of sexual development were used for RNA extraction (Fig. 1): (i) 4-week-old sclerotia prior to vernalization, (ii) primordia of stages 1 and 2 that were dissected from sclerotia, (iii) stipes of stages 3 and 4, (iv) apothecial disks of stages 5 and 6, and (v) pure ascospores obtained from mature apothecia. The samples were designated Scl, Apo12, Apo34, Apo56, and Asc, respectively. In addition, we sampled for RNA extraction the elongated stipes with a swelling (stage Apo34) from a cross between wild-type strain SAS405 and the *ΔMAT1-1-5* deletion mutant and from a cross between wild-type strain B05.10 and the *ΔMAT1-2-10* deletion mutant, which are defective in apothecial disk development (Fig. 2). Two biological replicates of each RNA sample were used for cDNA synthesis, quantitative real-time PCR (qRT-PCR), and RNA sequencing.

### Expression of mating type genes during development of apothecia.

**FIG 4:**
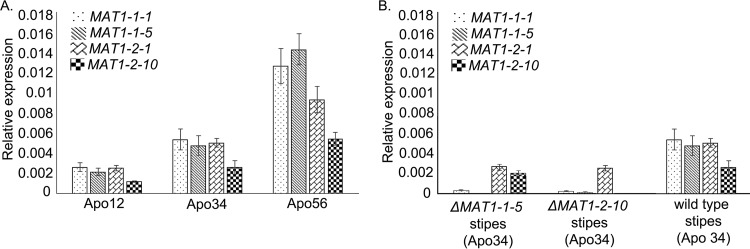
qRT-PCR analysis of *B. cinerea MAT* genes, relative to the internal standard *BctubA*. (A) Expression in three stages of apothecium development in a wild-type cross: primordia (left), stipes (middle), and disks (right). (B) Expression levels in mutant stipes (Δ*MAT1-1-5*, left; Δ*MAT1-2-10*, middle) compared to wild-type stipes in an equivalent stage (Apo34, right). Expression data from Apo34 wild-type stipes are the same between panels A and B.

First, qRT-PCR analysis was performed to determine the transcript levels of the *MAT1-1-1*, *MAT1-1-5*, *MAT1-2-1*, and *MAT1-2-10* genes over three stages of apothecium development (Apo12, Apo34, and Apo56) in a wild-type cross between SAS56 and SAS405. Transcript levels were normalized to the constitutively expressed β-tubulin gene *BctubA*. Figure 4A shows that the transcript levels of all four *MAT* genes were lowest in primordia (Apo12), slightly increased in stipes (Apo34), and peaked in apothecial disks (Apo56). Transcript levels of all *MAT* genes were also quantified in stipes defective in apothecial disk development, obtained from crosses between a wild-type strain and a *ΔMAT1-1-5* or *ΔMAT1-2-10* deletion mutant (Fig. 4B). In *ΔMAT1-1-5* stipes, the transcript of the *MAT1-1-5* gene was undetectable, as expected, but also transcript levels of the *MAT1-1-1* and *MAT1-2-1* genes were lower than in stipes from the cross between two wild-type isolates. In *ΔMAT1-2-10* stipes, the transcript of the *MAT1-2-10* gene was undetectable, but also the transcript levels of the other three *MAT* genes were lower than in stipes from the cross between two wild-type isolates. As discussed above, it is assumed that the mutant stipes failed to undergo karyogamy and therefore remained blocked in the dikaryotic stage. The expression profiles of *MAT* genes in the mutant stipes indicate that the absence of one *MAT* gene in a nucleus derived from one of the mating partners affects transcript levels of *MAT* genes in the nucleus derived from the opposite partner. How transcriptional cross talk between opposite *MAT* alleles in separate nuclei is accomplished remains to be studied.

### Whole-genome transcriptome analysis during development of apothecia.

RNA sequencing was performed on the five samples, each in two biological replicates. The library size and mapping efficiency of reads on the *B. cinerea* genome are listed in Table S1. Over 94% of the 11,700 gene models in the *B. cinerea* genome (17) were represented by at least one read in each individual sample; in every sample, at least 79% of all genes had counts per million (CPM) of ≥1. The transcriptome of sample Apo56 was the most diverse, with >90% of the gene models displaying CPM of ≥1. This sample also contained the greatest diversity of cell types, as material taken from apothecial disks was composed of cell layers of excipulum and hymenium, of dikaryotic cells, and of diploid cells undergoing meiosis as well as asci and ascospores in different stages of development and maturity. Sclerotia and ascospore samples, on the other hand, represent more homogeneous tissue types; it thus is logical that their expression profile was less diverse.

10.1128/mBio.01939-17.2 TABLE S1  Mapping efficiency of RNA-seq reads on the *Botrytis cinerea* (strain B05.10) genome. Five samples represent sclerotia (Scl), apothecial primordia (Apo12), apothecial stipes (Apo34), apothecial disks (Apo56), and ascospores (Asc); biological replicates are labeled A and B. The number and proportion of genes with CPM values of ≥1 are given. Download TABLE S1, XLSX file, 0.01 MB. Copyright © 2018 Rodenburg et al. 2018 Rodenburg et al. This content is distributed under the terms of the Creative Commons Attribution 4.0 International license.

### Expression of *B. cinerea* orthologs to genes involved in sexual development of model fungi.

Transcript levels of *B. cinerea* homologs to genes in *Aspergillus nidulans* and *Sordaria macrospora* that were previously listed as activators or repressors of sexual development in these fungi were examined (51, 52). Blast searches identified 88 *B. cinerea* homologs for 70 of these *A. nidulans* and *S. macrospora* genes (Table S2). Expression of these *B. cinerea* genes was detectable in all samples tested, with CPM values ranging from 0.1 to 6,282. Table S2 presents the expression profiles of the *B. cinerea* genes, grouped on the basis of their proposed role (in *A. nidulans*) in perception of environmental signals, mating processes and signal transduction, transcription factors, endogenous physiological processes, and ascospore production and maturation, according to the work of Dyer and O’Gorman (51). Expression profiles of a subset of these genes are discussed below in more detail. The *Bcwcl2* gene and the *Bcvel1* gene are putative positive regulators of sexual reproduction, and their transcript levels increased 2- to 3-fold in *B. cinerea* primordia and mature stipes, compared to sclerotia and ascospores, while transcripts of *Bcwcl1* and *Bcvel2* genes were less abundant and barely changed during sexual development. Genes encoding components of the *B. cinerea* heterotrimeric G protein complex (Gα1, Gβ, and Gγ but not Gα2 and Gα3) showed slight increases in transcript levels in apothecial tissues compared to sclerotia and ascospores. Furthermore, the G protein signaling regulator and a phosducin-like chaperone showed ~8-fold increases in transcript levels at the transition from sclerotia to apothecial primordia. The transcript levels of transcription factor genes *BcnsdD*, *BcfhpA*, and *BcnrdA*/*msnA* (positive regulators of sexual reproduction in *A. nidulans*) increased strongly in all stages of apothecial development and decreased in ascospores, while expression patterns of *BcflbC* and *BcstuA* (negative regulators of sexual reproduction in *A. nidulans*) showed an almost inverse pattern. The expression profiles of *B. cinerea* genes orthologous to *S. macrospora* genes required for sexual development (52) were also examined (Table S2). The transcript level of the gene encoding ATP citrate lyase subunit ACL1 increased 3-fold in the transition from sclerotia to primordia, remained constant in apothecia, and further increased in ascospores. The ortholog of *S. macrospora pro4*/*leu1* (β-isopropylmalate dehydrogenase) was expressed abundantly in primordia and stipes but at low levels in sclerotia, apothecial disks, and ascospores; *BcnoxD*, the ortholog of *S. macrospora pro41*, was highly expressed during all stages of apothecial development but at low levels in sclerotia and ascospores.

10.1128/mBio.01939-17.3 TABLE S2  Transcript levels of *Botrytis cinerea* orthologs to genes in *Aspergillus nidulans* and *Sordaria macrospora* previously listed as activators or repressors of sexual development (51, 52). The *A. nidulans* and *S. macrospora* gene symbols, locus identifier, and their proposed functions and domains are given in columns A to C; whether those genes are considered activators or repressors of sexual development is indicated in column D, with the relevant reference provided in column E. Gene identifiers (IDs) for the *B. cinerea* orthologs are given in column F, with their expression values in the five tissue samples (in counts per million mapped reads [CPM]) presented in columns G to K as averages from two biological replicates. Download TABLE S2, XLSX file, 0.03 MB. Copyright © 2018 Rodenburg et al. 2018 Rodenburg et al. This content is distributed under the terms of the Creative Commons Attribution 4.0 International license.

In summary, a number of *B. cinerea* genes which, based on functions described in *A. nidulans*, likely act as positive regulators of sexual development had high transcript levels in early stages of apothecium development, which dropped in mature apothecial disks and ascospores. Conversely, several *B. cinerea* genes that, based on functions described in *A. nidulans*, likely act as negative regulators of sexual development had high transcript levels in sclerotia and primordia, which dropped in later stages of apothecium development and ascospores. Although such patterns would be in agreement with proposed positive or negative regulatory roles of these genes in sexual development, validation of their role in sexual reproduction would require functional analysis. Such expression patterns were, however, not observed for all *B. cinerea* genes that (based on functions in *A. nidulans*) might act as positive or negative regulators in sexual development. In several cases, the expression level was constant or showed a distinct pattern. It should, in this context, be considered that the role of a particular gene as a regulator of sexual development does not necessarily imply that such a gene is differentially transcribed during sexual development.

### Genes differentially expressed between consecutive stages of sexual development.

Changes in gene expression during developmental transitions were analyzed by comparing expression levels between subsequent stages of apothecial development (Scl and Apo12, Apo12 and Apo34, Apo34 and Apo56, and Apo56 and Asc). Differentially expressed genes [adjusted *P* value of <0.05 and log_2_(fold change [FC]) of >2 or <−2) that were identified are recorded in Table 2 and listed in Tables S3 to S6. Pronounced changes in transcript levels were detected in the transition from sclerotia to primordia (>2,500 differentially expressed genes) and even more so between apothecial disks and ascospores, in which >3,900 genes were differentially expressed (i.e., one-third of the 11,700 genes in the *B. cinerea* genome). In contrast, only 75 genes were differentially expressed between primordia and stipes, whereas between stipes and apothecial disks, the number of differentially expressed genes was 594.

10.1128/mBio.01939-17.4 TABLE S3  Differentially expressed genes in the transition from sclerotia to apothecial primordia. Panels S3a and S3b list the upregulated and downregulated genes, respectively; their expression values in both biological replicates of both samples (in counts per million mapped reads [CPM]); the log_2_ of the fold change (logFC); average log_2_ of the CPM values between the four RNA samples (logCPM); test statistic of the likelihood-ratio test (LR); the uncorrected and Benjamini-Hochberg-corrected *P* value of the likelihood test; the *B. cinerea* gene name; and a description resulting from manual annotation. Panels S3c and S3d present GO enrichment analyses for the upregulated and downregulated genes, respectively. Download TABLE S3, XLSX file, 0.5 MB. Copyright © 2018 Rodenburg et al. 2018 Rodenburg et al. This content is distributed under the terms of the Creative Commons Attribution 4.0 International license.

**TABLE 2:** Differentially expressed genes during developmental transitions

Comparison	No. of genes	
	Upregulated [log_2_(FC) > 2]	Downregulated [log_2_(FC) <− 2]
Scl vs Apo12	1,570	1,011
Apo12 vs Apo34	37	38
Apo34 vs Apo56	318	276
Apo56 vs Asc	1,424	2,485

**FIG 5:**
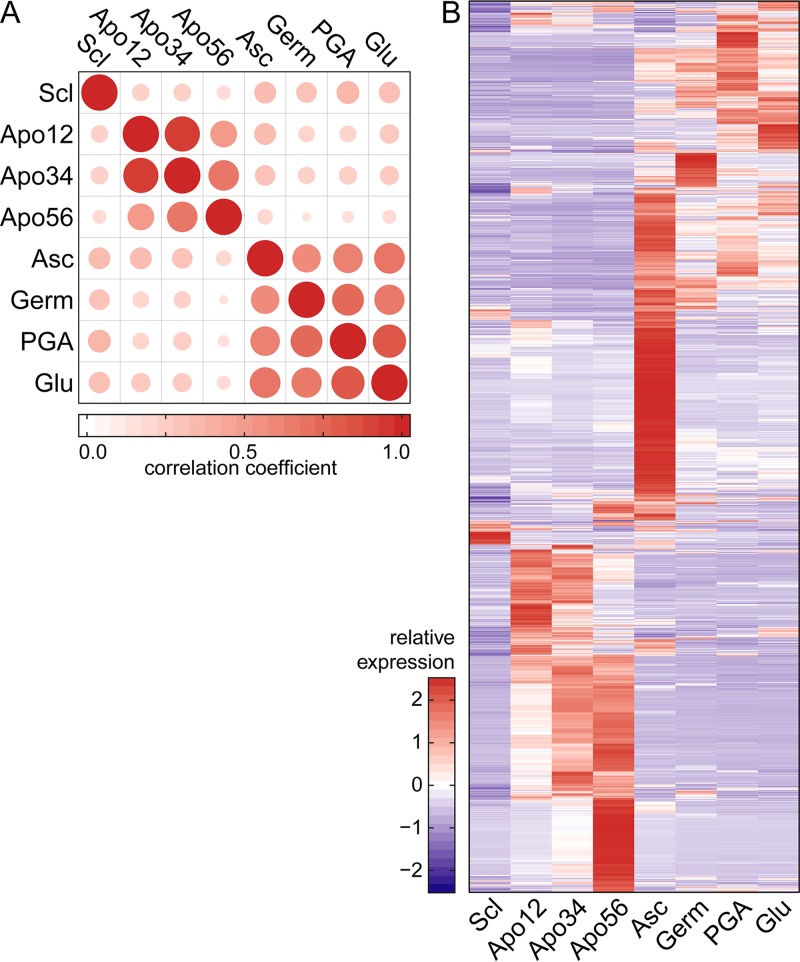
Comparison of transcriptomes of five stages of sexual development with three asexual tissues (Germ, germinating conidia [53]; PGA, mycelium on polygalacturonate-containing medium [54]; Glu, mycelium on glucose-containing medium [54]). For each gene in these comparisons, the mean CPM was calculated over available replicates. (A) Pairwise correlations between CPM values of *B. cinerea* total transcriptome samples. Dot size and color represent the Pearson correlation coefficient. (B) Relative expression (*Z*-scores) of subset of 3,084 genes that were differentially expressed in at least one of the sexual development stages (Scl, Apo12, Apo34, Apo56, and Asc) compared to asexual tissues.

In order to examine whether the expression patterns during sexual development were distinct from asexual development, we determined pairwise correlations between CPM values in the five stages of sexual development reported here and three published data sets of asexual tissues, representing RNA from young germlings (conidia inoculated in liquid medium and grown for 12 h) (53) or from mycelium grown in polygalacturonate or glucose (54) (Fig. 5A). The correlations between samples were generally low (<0.5) with some exceptions. The transcriptomes in stages Apo12 and Apo34 were very similar to one another, and these tissues are morphologically alike. All other pairwise correlations between sexual and asexual stages were <0.75, with sclerotia displaying the most dissimilar expression profile.

The expression levels of all 3,084 genes that are differentially expressed during at least one of the five stages of sexual development were plotted in a heat map and compared to levels in the asexual tissues. The heat map (Fig. 5B) shows that the expression patterns of this gene set differed markedly between each of the sexual stages and the three nonsexual stages. Features of the sets of differentially expressed genes are discussed below. Gene ontology (GO) enrichment analysis was carried out on the four sets of differentially expressed genes (adjusted *P* value of <0.05, any fold change).

### Differentially expressed genes in the transition from sclerotia to primordia.

The transition from sclerotia to primordia was associated with significantly increased transcript levels of 1,570 genes and reduced transcript levels of 1,011 genes (Table S3). A major change in secondary metabolism was observed. Among the upregulated genes (Table S3a) were 10 distinct secondary metabolite (SM) gene clusters, involved in production of polyketides (6 clusters), sesquiterpenes (2 clusters), and nonribosomal peptides (2 clusters), for all of which the SMs that they produce are unknown. Concomitantly, there was downregulation (Table S3b) of 5 SM clusters involved in the production of polyketides (2 clusters) and nonribosomal peptides (3 clusters). Among the upregulated genes (Table S3a) were also several regulatory genes, such as the *MAT1-1-1* and *MAT1-1-5* genes, two homeobox genes (*Bchox4* and *Bchox8* [55]), the Velvet complex subunit *Bcvel4*, the Vivid-like putative light sensor, the Medusa-like transcriptional regulator, and the light-responsive GATA-type transcription factor *Bcltf1* (56); several genes involved in histone modification and DNA methylation (see below); hydrophobin genes *Bhp1*, *Bhp2*, and *Bhl1* (57); five laccase genes; and 19 transporter genes of various classes. Among the downregulated genes (Table S3b) were, besides genes in five SM clusters mentioned above, regulatory genes such as HMG-box transcription factor-encoding gene *Bcste11* (58) and the mitogen-activated protein (MAP) kinase gene *Bmp3* (59), several genes involved in histone modification (see below), four glutathione *S*-transferases, the hydrophobin gene *Bhp3*, the melanin biosynthetic tetrahydroxynaphthalene reductase gene *Bcbrn1* (60, 61), the sclerotium-specific lectin gene *Bcssp1*, and 28 transporters of various classes.

GO term enrichment analysis on the upregulated genes showed overrepresentation of (among others) the terms ribosome biogenesis, DNA replication, microtubule-based movement, fatty acid biosynthetic process, lipid biosynthetic process, oxidation-reduction process, tricarboxylic acid cycle, and aerobic respiration (Table S3c). GO term enrichment analysis on the downregulated genes showed an overrepresentation of (among others) the terms oxidation-reduction process, hexose metabolic process, and transmembrane transport (Table S3d).

### Differentially expressed genes in the transition from primordia to stipes.

The transition from primordia (Apo12) to stipes (Apo34) was associated with significantly different transcript levels of 75 genes (Tables 2 and S4a and b). Among the 38 upregulated genes were 13 genes (34%) of unknown function that are specific to *B. cinerea* or the family *Sclerotiniaceae*. Among the 37 downregulated genes were a gene (Bcin04g02570) encoding a small secreted cysteine-rich protein (86-amino-acid mature protein with 13 Cys residues of which four occur in two Cys–Cys pairs) that does resemble, but not precisely match, the pattern for hydrophobin-like proteins; two genes encoding glycosyltransferases (CAZY GT2 and GT4 family proteins); and two genes in an SM gene cluster comprising polyketide synthase gene *Bcpks15*, of which the product is unknown. GO term enrichment analysis detected overrepresentation of the term response to stress in the set of upregulated genes (Table S4c). GO term enrichment analysis on the set of downregulated genes (Table S4d) showed an overrepresentation of (among others) the terms ribosome biogenesis, rRNA processing, and oxidation-reduction process.

10.1128/mBio.01939-17.5 TABLE S4  Differentially expressed genes in the transition from apothecial primordia to stipes. Panels S4a and S4b list the upregulated and downregulated genes, respectively; their expression values in both biological replicates of both samples (in counts per million mapped reads [CPM]); the log_2_ of the fold change (logFC); average log_2_ of the CPM values between the four RNA samples (logCPM); test statistic of the likelihood-ratio test (LR); the uncorrected and Benjamini-Hochberg-corrected *P* value of the likelihood test; the *B. cinerea* gene name; and a description resulting from manual annotation. Panels S4c and S4d present GO enrichment analyses for the upregulated and downregulated genes, respectively. Download TABLE S4, XLSX file, 0.4 MB. Copyright © 2018 Rodenburg et al. 2018 Rodenburg et al. This content is distributed under the terms of the Creative Commons Attribution 4.0 International license.

### Differentially expressed genes in the transition from stipes to apothecial disks.

The transition from stipes (Apo34) to apothecial disks (Apo56) was associated with significantly increased transcript levels of 318 genes and significantly reduced transcript levels of 276 genes (Tables 2 and S5). Among the upregulated genes (Table S5a) were the gene encoding the activator of meiotic anaphase-promoting complex *Bcama1*; the hydrophobin gene *Bhp3* (57); several transporter-encoding genes, including the aquaporin genes *Bcaqp2* and *Bcaqp5* (62), the ABC transporter gene *Bmr3*, and hexose transporter genes *Bchxt2*, *Bchxt5*, and *Bchxt9* (63); and a bZIP transcription factor gene. Among the downregulated genes (Table S5b) were the endopolygalacturonase gene *Bcpg6*, two nonribosomal peptide synthase genes (*Bcnrps5* and *Bcnrps8*), the oxaloacetate hydrolase gene *Bcoah1* (64), the gene encoding the transcriptional regulator of sexual development *nosA* (ortholog of *S. macrospora pro1* [52]), the aspartyl proteinase gene *Bcap9* (65), the laccase gene *Bclcc10*, the DNA repair protein RAD1-encoding gene, the ceratoplatanin gene *Bcspl1* (66), and the sclerotium-specific lectin gene *Bcssp1*.

10.1128/mBio.01939-17.6 TABLE S5  Differentially expressed genes in the transition from apothecial stipes to disks. Panels S5a and S5b list the upregulated and downregulated genes, respectively; their expression values in both biological replicates of both samples (in counts per million mapped reads [CPM]); the log_2_ of the fold change (logFC); average log_2_ of the CPM values between the four RNA samples (logCPM); test statistic of the likelihood-ratio test (LR); the uncorrected and Benjamini-Hochberg-corrected *P* value of the likelihood test; the *B. cinerea* gene name; and a description resulting from manual annotation. Panels S5c and S5d present GO enrichment analyses for the upregulated and downregulated genes, respectively. Download TABLE S5, XLSX file, 0.1 MB. Copyright © 2018 Rodenburg et al. 2018 Rodenburg et al. This content is distributed under the terms of the Creative Commons Attribution 4.0 International license.

GO term enrichment analysis on the set of upregulated genes showed an overrepresentation of (among others) the terms cellular chemical homeostasis, amino acid transmembrane transport, heme metabolic process, oxidation-reduction process, cellular biogenic amine metabolic process, dephosphorylation, carbohydrate metabolic process, autophagy, organic anion transport, phosphatidylinositol metabolic process, and aspartate family amino acid biosynthetic process (Table S5c). GO term enrichment analysis on the set of downregulated genes showed an overrepresentation of (among others) the terms oxidation-reduction process, carbohydrate metabolic process, potassium ion transport, sterol metabolic process, and response to oxidative stress (Table S5d).

### Gene expression in mutant stipes blocked in transition to the apothecial disk.

The transcriptomes of mutant stipes, obtained in crosses with *ΔMAT1-1-5* and *ΔMAT1-2-10* deletion mutants, were determined by RNA-seq and compared to one another and to the transcriptome of wild-type stipes at the equivalent stage (Apo34). A total of 1,310 genes were differentially expressed between mutant stipes from the SAS405 × *ΔMAT1-1-5* mutant cross and stipes from a wild-type cross (Tables 3 and S6). Among this total set, 854 genes were downregulated and 456 genes were upregulated in the mutant stipes (blocked in transition to the apothecial disk), compared to stipes from the cross between two wild-type strains (Table S6a and b). In the cross between wild-type strain B05.10 and the *ΔMAT1-2-10* mutant, 985 genes were downregulated and 152 genes were upregulated in mutant stipes, compared to stipes in a cross between wild-type strains (Table S6c and d).

10.1128/mBio.01939-17.7 TABLE S6  Differentially expressed genes in stipes obtained with *MAT* gene mutants. Panels S6a and S6b list the downregulated and upregulated genes, respectively, in stipes obtained in an SAS405 × *ΔMAT1-1-5* cross, compared to stipes from a wild-type cross, SAS405 × SAS56. Panels S6c and S6d list the downregulated and upregulated genes, respectively, in stipes obtained in an SAS56 × *ΔMAT1-2-10* cross, compared to stipes from a wild-type cross, SAS405 × SAS56. Panel S6e lists the overlap between downregulated genes in panels S6a and S6c, while panel S6f lists the overlap between upregulated genes in panels S6b and S6d. Download TABLE S6, XLSX file, 0.3 MB. Copyright © 2018 Rodenburg et al. 2018 Rodenburg et al. This content is distributed under the terms of the Creative Commons Attribution 4.0 International license.

**TABLE 3:** Differentially expressed genes in stipes of *MAT* gene mutants compared to wild-type stipes

Comparison	No. of genes	
	Downregulated [log_2_(FC) > 2]	Upregulated [log_2_(FC) <− 2]
Δ*MAT1-1-5* vs wild type	854	456
Δ*MAT1-2-10* vs wild type	985	152

There was some overlap between the differentially expressed genes in both mutant stipes: compared to wild-type stipes, 87 genes were downregulated in both the SAS405 × *ΔMAT1-1-5* cross and the B05.10 × *ΔMAT1-2-10* cross, while 26 genes were upregulated in both mutant stipes (Table S6e and f). This list of 87 genes was further compared with the genes that are, in crosses between two wild-type isolates, upregulated in the transition from stipes (stage Apo34) to mature apothecial disks (stage Apo56). Among the genes that were not upregulated in both mutant stipes while they were upregulated in wild-type apothecia were one Zn_2_Cys_6_ transcription factor-encoding gene, four major facilitator superfamily (MFS) transporter genes, the *MAT1-1-1* and *MAT1-2-1* genes, a HIT/MYND domain containing gene, and a G-protein-coupled receptor (BcGPCR2), as well as 24 genes of unknown function that are specific either to *Botrytis* or to the family *Sclerotiniaceae*.

### Differentially expressed genes in the transition from apothecial disks to ascospores.

Between the apothecial disks (Apo56) and the ascospore sample, as many as 3,909 genes were differentially expressed, of which 37% were upregulated and 63% were downregulated (Tables 2 and S7a and b). Among the upregulated genes were 57 genes encoding ribosomal proteins and 24 other genes involved in ribosome biogenesis, as well as 10 translation initiation and elongation factor-encoding genes and 9 mitochondrial protein-encoding genes. The gene set also contains the *Bcnop1* gene, encoding a histone glutamine methyltransferase; the heterotrimeric G protein β subunit gene *Bcgbl1*; and five calcineurin-dependent genes (67) as well as the calcineurin regulator calcipressin gene (68). GO term enrichment analysis on the set of upregulated genes showed an overrepresentation of >200 terms (Table S7c), while GO term enrichment analysis on the downregulated genes showed an overrepresentation of 42 terms (Table S7d). A substantial number of genes upregulated in ascospores were of special interest, despite not being reflected in the GO enrichment analysis by lack of annotation in the “biological process” domain. These genes are discussed in more detail below.

10.1128/mBio.01939-17.8 TABLE S7  Differentially expressed genes in the transition from apothecial disks to ascospores. Panels S7a and S7b list the upregulated and downregulated genes, respectively; their expression values in both biological replicates of both samples (in counts per million mapped reads [CPM]); the log_2_ of the fold change (logFC); average log_2_ of the CPM values between the four RNA samples (logCPM); test statistic of the likelihood-ratio test (LR); the uncorrected and Benjamini-Hochberg-corrected *P* value of the likelihood test; the *B. cinerea* gene name; and a description resulting from manual annotation. Panels S7c and S7d present GO enrichment analyses for the upregulated and downregulated genes, respectively. Download TABLE S7, XLSX file, 1 MB. Copyright © 2018 Rodenburg et al. 2018 Rodenburg et al. This content is distributed under the terms of the Creative Commons Attribution 4.0 International license.

### Ascospores are transcriptionally primed for host plant invasion.

Inspection of the list of genes upregulated in ascospores revealed numerous genes that encode enzymes potentially contributing to plant invasion, such as components of the fungal pectinolytic machinery: endopolygalacturonase genes *Bcpg2* and *Bcpg6* (69); the pectin methylesterase gene *Bcpme2* (70); the galacturonate reductase gene *Bcgar2* (48); aspartyl proteinase genes *Bcap3*, *Bcap4*, and *Bcap14* (65); laccase genes *Bclcc2* and *Bclcc8*, the former encoding an enzyme that can oxidize the grapevine phytoalexin resveratrol (71); five ABC multidrug efflux transporter-encoding genes, *BcatrA* (72), *Bmr1* (73), *BcatrO* (74), *BcatrD* (75), and *BcatrB*, involved in the tolerance of *B. cinerea* to fungicides and plant defense compounds (72, 76), as well as the Zn_2_Cys_6_ transcription factor *Bcmrr1*, which regulates *BcatrB* expression (77); and genes encoding proteins involved in signal transduction pathways—the heterotrimeric G protein subunit Gβ1, a phosphoinositide biosynthetic gene, the regulator of calcineurin (calcipressin) (68), the two-component histidine kinase *Bos1* (78, 79), and the G-protein-coupled receptor *Bcgpr1*. The list also comprises five hexose transporter-encoding genes (63), the oxalic acid biosynthetic gene *Bcoah1* (64), the superoxide dismutase gene *Bcsod3*, and six CND (calcineurin-dependent) genes (67). Finally, several genes encoding chromatin-modifying enzymes were also upregulated, such as the histone glutamine methyltransferase gene *Bcnop1*, the histone deacetylase gene *Bchst2*, and the histone chaperone gene *Bcnap1* (see below).

Among the genes that were downregulated between mature apothecial disks and ascospore samples were several genes encoding histone-modifying enzymes and C-5 cytosine-specific DNA methylases (see below), as well as the Dicer-like gene *Bcdcl1* (80); as many as 15 SM clusters (six nonribosomal peptide synthase [NRPS] clusters, six polyketide synthase [PKS] clusters, one sesquiterpene synthase [STC] cluster, the PHS1 carotenoid cluster, and the YGH1 cluster involved in melanin biosynthesis); the homeobox gene *Bchox6* (53); regulators of sexual development such as the *MAT1-1-1* gene, the NADPH oxidase gene *BcnoxD* (ortholog of *S. macrospora pro41* [81]), the transcriptional regulator *nosA* (ortholog of *S. macrospora pro1*), and the *Bcspo11* gene, encoding a protein that initiates meiotic recombination; the opsin gene *Bop1*; six laccase genes; and three aquaporin genes, *Bcaqp5*, *Bcaqp8*, and *Bcaqp9* (62).

### Spatially clustered coregulation of transcription during sexual development.

**FIG 6:**
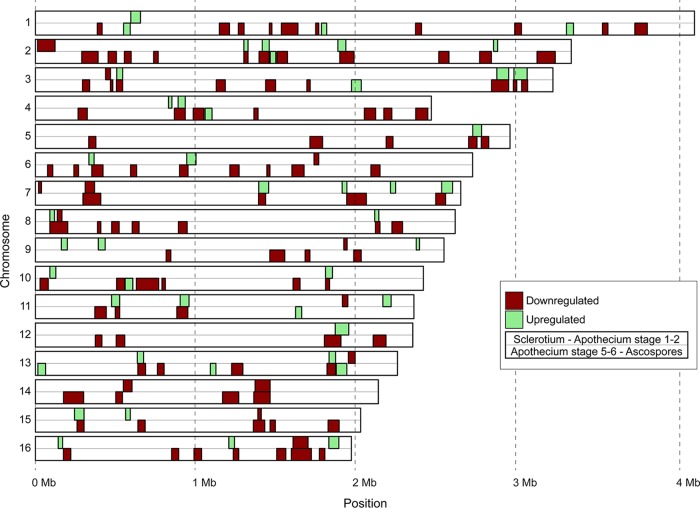
CROC analysis for identification of clusters of coregulated genes. Core chromosomes of *B. cinerea* strain B05.10 are displayed vertically with Chr1 at the top and Chr16 at the bottom. Size markers are provided at the bottom. Clusters of upregulated genes are displayed in green, while clusters of downregulated genes are displayed in red. For each chromosome, two rows are provided: the top row displays clusters of genes coregulated during the transition from sclerotia to primordia, while the bottom row displays clusters of genes coregulated during the transition from apothecial disks to ascospores. Minichromosomes 17 and 18 (17) did not contain coregulated clusters and were omitted for simplicity.

Using sliding windows of ≥12 genes, more than 150 cases were observed in which at least 50% of genes were significantly coregulated (either jointly up- or jointly downregulated) during the same developmental transition (Table 4). At the onset of sexual development, during the transition from sclerotia to primordia, 35 coregulated clusters were identified with an average length of 44.7 kb and containing on average 8 upregulated genes. Even more notably, there were 99 clusters of downregulated genes at the completion of sexual reproduction (the transition from apothecial disks to ascospores), with an average length of 56.5 kb and containing on average 9.3 downregulated genes (Table 4; Fig. 6). The largest of these clusters was 135 kb in length and contained as many as 24 genes. Altogether, these 99 clusters contained 923 genes (Table S8), representing 37% of the 2,486 genes that were downregulated during the transition from apothecial disks to ascospores. Of the 35 clusters that were upregulated in the transition from sclerotia to primordia, 23 (66%) overlapped at least partially with the 99 clusters that were downregulated in ascospores, which was especially notable in chromosomes 2, 3, and 13 (Fig. 6).

10.1128/mBio.01939-17.9 TABLE S8  CROC analysis for identification of clusters of coregulated genes. Clusters were defined as sliding windows of ≥12 genes, of which at least 50% are jointly upregulated or downregulated. Each table provides information about the chromosome number, the start and end coordinates of the cluster, the adjusted *P* value for clustering (threshold was set at a *P* value of <0.05), and the gene IDs of the coexpressed genes in the cluster. CROC output is provided for upregulated genes in the transition from sclerotia to primordia (panel S8a), downregulated genes in the transition from sclerotia to primordia (panel S8b), downregulated genes in the transition from primordia to stipes (panel S8c), upregulated genes in the transition from apothecial disks to ascsopores (panel S8d), and downregulated genes in the transition from apothecial disks to ascsopores (panel S8e). Download TABLE S8, XLSX file, 0.03 MB. Copyright © 2018 Rodenburg et al. 2018 Rodenburg et al. This content is distributed under the terms of the Creative Commons Attribution 4.0 International license.

**TABLE 4:** Clustering of coregulated genes during transitions in fruiting body development^a^

Comparison	No. of genes	
	Upregulated	Downregulated
Scl vs Apo12	35	13
Apo12 vs Apo34	0	0
Apo34 vs Apo56	0	2
Apo56 vs Asc	11	99

a Clusters are defined as blocks of at least 12 consecutive genes, of which at least 50% show simultaneous up- or downregulation.

**FIG 7:**
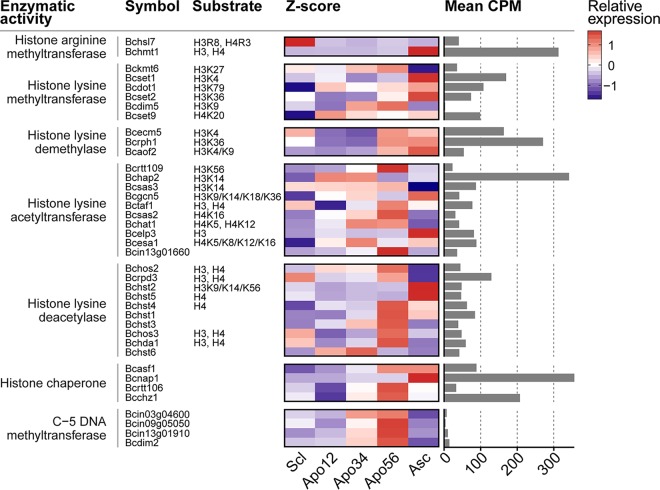
Transcript abundance of enzyme-encoding genes involved in histone modification and DNA methylation, at five stages in apothecium development. Columns from left to right indicate the respective annotated enzymatic activity, assigned gene symbol, the modified residue on the histone, the *Z*-score-transformed expression profiles over the five determined life stages, and the mean CPM values over the five stages to relate the relative changes to absolute, respectively. Functional annotations are taken from reference 95.

The spatial pattern in transcriptional changes, especially during the onset and the end of sexual development, suggested the occurrence of changes in chromatin organization during the developmental transitions in apothecium development. The importance of histone acetylation and chromatin remodeling in meiotic recombination was reported in the fission yeast, *Schizosaccharomyces pombe* (82). Furthermore, the histone chaperone ASF1 in *Sordaria macrospora* was shown to be essential for the formation of mature perithecia (83). We examined the transcriptional profiles of 39 genes encoding histone-modifying enzyzmes or DNA methylases in the five stages of sexual development (Fig. 7). Half of these genes showed the highest expression level in the mature apothecia, in which meiosis was taking place. Notably, all four genes encoding C-5 cytosine-specific DNA methyltransferase and six of the 10 histone lysine deacetylase genes showed a clear peak of expression in this sample. In contrast, genes encoding two histone lysine methyltransferases, a histone lysine acetyltransferase, and two other histone lysine deacetylases, as well as a histone chaperone, displayed a strong peak of expression in the ascospores. Finally, one histone arginine methyltransferase gene was predominantly expressed in sclerotia. Chromatin immunoprecipitation sequencing (ChIP-seq) and bisulfite sequencing experiments in the different stages of *B. cinerea* apothecium development will be required to explore the changes in chromatin architecture during these developmental transitions. The roles of histone-modifying enzymes and DNA methylases in sexual reproduction can be validated by targeted mutagenesis (if such mutants are viable).

### Conclusion.

This study presents the first transcriptional analysis of sexual development in the *Helotiales* and a unique view on the transcriptome of pure ascospores of a filamentous ascomycete. The comparison of expression profiles of consecutive stages of sexual development revealed massive changes in transcript levels in the transition from sclerotia to primordia (the onset of sexual development) and even more so in the transition from mature disks to ascospores (completion of sexual reproduction). The number and nature of genes that were upregulated in the ascospores indicate that these spores are transcriptionally primed for the production of virulence factors prior to their first encounter with a host plant. *B. cinerea* ascospores thus do not land on plant tissues totally naive but possess a reservoir of virulence factors at their arrival, which facilitates a fast and effective invasion. Such an observation does not preclude that virulence genes might be further upregulated during germination of the ascospores on host tissue. It will be interesting to study whether the induction of virulence genes also occurs during development of asexual conidia, which are as infectious as ascospores and form important propagules for dispersal of *B. cinerea* to neighboring host plants. The present study also provided evidence for a clear spatial clustering in transcriptional changes during the onset and termination of sexual development, which merits further studies on epigenetic changes during *B. cinerea* apothecium development.

## MATERIALS AND METHODS

### Strains.

Wild-type *B. cinerea* strains SAS56 (reference *MAT1-1* strain) and SAS405 (reference *MAT1-2* strain) were used (18, 84), as well as strain B05.10 (*MAT1-1*), for which a gapless reference genome sequence was determined (17). Strains were plated on malt extract agar (MEA; Oxoid, Basingstoke, United Kingdom). For obtaining sclerotia, cultures were grown at 15°C in darkness. For obtaining conidia, cultures were grown at 20°C for 3 to 4 days and then exposed for 1 day to near-UV light and incubated for another week before conidiospores were collected.

### Deletion of mating type genes.

The *MAT1-1-1*, *MAT1-1-5*, *MAT1-2-1*, and *MAT1-2-10* genes were individually targeted for mutagenesis by homologous recombination (see Fig. S1). *MAT1-1-1* and *MAT1-1-5* genes were deleted in SAS56, whereas the *MAT1-2-1* and *MAT1-2-10* genes were deleted in SAS405. In addition, the *MAT1-1-5* gene was deleted in B05.10. The strategies for generating deletion constructs, *B. cinerea* protoplast transformation, and PCR-based screening of transformants were as described previously (70). Table S9 contains primers that were used for amplification of gene deletion fragments. The hygromycin resistance (HPH) cassette, amplified from vector pLOB7 (48) with primer pair Cassette-5/Cassette-3, was used as a selection marker. Genomic DNA of transformants was analyzed for the presence/absence of the wild-type target gene by amplifying the target genes *MAT1-1-1*, *MAT1-1-5*, *MAT1-2-1*, and *MAT1-2-10* using primer combinations MAT111-5.1/MAT111-3.1, MAT115-5.1/MAT115-3.1, MAT121-5.1/MAT121-3.1, and MAT124-5.1/MAT124-3.1, respectively.

10.1128/mBio.01939-17.10 TABLE S9  Primers used in this study. Download TABLE S9, XLSX file, 0.01 MB. Copyright © 2018 Rodenburg et al. 2018 Rodenburg et al. This content is distributed under the terms of the Creative Commons Attribution 4.0 International license.

### DNA extraction.

Freeze-dried mycelium was used for DNA extraction, using a Gentra Puregene DNA purification kit (Qiagen, Venlo, The Netherlands), according to manufacturer’s instructions.

### Sexual crosses.

Sexual crosses were performed using wild-type strains (SAS56, SAS405, and B05.10) and four mutants: the *ΔMAT1-1-1*, *ΔMAT1-1-5*, *ΔMAT1-2-1*, and *ΔMAT1-2-10* strains. Crosses were set up using previously described protocols (18, 19). Sclerotia (incubated at 0°C for 1 month) were sampled from MEA plates, and their surfaces were cleaned with water using a soft toothbrush. Three to five sclerotia of ~1 cm in size were placed in single wells in a six-well microtiter plate. Suspensions of microconidia and hyphal fragments were prepared by pouring sterile water onto the plate from which sclerotia were removed, followed by gentle rubbing with a plastic spatula. Sclerotia in the microtiter plate were fertilized with this suspension (3 ml per well). The plate was sealed with Parafilm and incubated at 12°C in candescent light with a 12-h photoperiod. Apothecia developed over a period of 60 to 90 days. Ascospores were sampled from mature apothecia by gently crushing apothecial disks in water and filtering the suspension over glass wool to separate hymenium tissue and large debris from the ascospores (in the flowthrough). The suspension of ascospores was centrifuged at 250 × *g* for 10 min, and the pellet was resuspended in water for rinsing and centrifuged again.

### RNA extraction and RNA sequencing.

Samples of three different developmental stages of apothecia, as well as of ascospores, were freeze-dried, and total RNA was isolated using the Nucleospin RNA plant kit (Macherey-Nagel, Düren, Germany), according to the manufacturer’s instructions. For isolation of RNA from sclerotia, freeze-dried sclerotia were frozen in liquid nitrogen and ground with a mortar and pestle. Total RNA was isolated with TRIzol reagent (Life Technologies, Grand Island, NY, USA) in combination with the Nucleospin RNA plant kit, according to manufacturer’s instructions. Integrity of RNA was monitored by gel electrophoresis, and the concentration determined using a NanoDrop 2000 spectrophotometer (Thermo Scientific, Wilmington, DE, USA). All samples were collected in two biological replicates. Twenty micrograms of RNA from each sample was used by the Beijing Genome Institute (Hong Kong, China) for library construction (300- to 700-nucleotide [nt] insert sizes) and sequenced using an Illumina HiSeq 2000 platform. Paired-end RNA-seq reads of 90 nt were assessed for quality using FastQC v0.10.1 (Babraham Institute; http://www.bioinformatics.babraham.ac.uk/projects/fastqc). Reads were trimmed to 80 nt using FASTX-Toolkit v0.13.2 (http://hannonlab.cshl.edu/fastx_toolkit).

### cDNA synthesis and qRT-PCR.

First-strand cDNA was synthesized from 1 µg total RNA with Moloney murine leukemia virus (MMLV) reverse transcriptase (Promega, Leiden, The Netherlands) according to the manufacturer’s instructions. Quantitative real-time PCR (qRT-PCR) was performed using an ABI7300 PCR machine (Applied Biosystems, Foster City, CA, USA) in combination with the qPCR SensiMix kit (Bioline, London, United Kingdom) using primers listed in Table S9. qRT-PCR conditions were as follows: an initial 95°C denaturation step for 10 min, followed by denaturation for 15 s at 95°C and annealing/extension for 1 min at 60°C, for 40 cycles. The data were analyzed on the 7300 System SDS software (Applied Biosystems, Foster City, CA, USA). The gene expression values were normalized to the *B. cinerea* tubulin gene, *BctubA* (85).

### Read mapping and quantification of expression.

RNA-seq reads of the five stages of sexual development and three asexual tissues (53, 54) were trimmed and mapped onto the *Botrytis cinerea* B05.10 genome (17) using HISAT v2.0.3-beta (86). The mapping efficiency ranged from 90.5 to 95.9% (Table S1). Expression was quantified using Rsubread v1.16.1 (87), by considering reads that were fully aligned to exons, based on the manually curated genome annotations (http://fungi.ensembl.org/Botrytis_cinerea). Reads overlapping multiple exons were also counted multiple times. Consecutively, genes expressed at low levels (i.e., read counts of <10 in all samples) were filtered. The read counts were normalized by using the trimmed mean of M-values method from the EdgeR package v3.8.6 deriving counts per million (CPM) read values (88, 89). Subsequently, differential gene expression between pairs of consecutive RNA-seq samples was assessed by fitting generalized linear models (GLMs) to the expression data under a negative binomial distribution, weighting count values for replicate effects and sample dispersion. Resulting *P* values were corrected for multiple testing using the Benjamini-Hochberg method (90). The functional annotations of genes with a log_2_(FC) of >2 (or <−2) between two samples, at an adjusted *P* value of <0.05, were manually inspected. For visual inspection and comparison of expression profiles, the CPM expression values were scaled using a *Z*-score transformation: *Z* = (*X* − μ)/σ. Here, *X* is the CPM value of the respective gene at a particular stage, and μ and σ represent the mean and standard deviation for this gene over the five stages, respectively.

### Enrichment tests.

To test for overrepresented gene ontology (GO) terms in each set of differentially expressed genes, GO enrichment analysis was performed. The total set of proteins from *B. cinerea* was annotated with GO terms using InterProScan v5.18.57 (91). Using the R package TopGO v2.18.0 (92), Fisher’s exact tests were performed on the GO terms associated with the significantly up- and downregulated genes (adjusted *P* value of <0.05) between every consecutive pair of conditions. The resulting lists of significantly overrepresented GO terms were trimmed using REVIGO (93).

### Differentially expressed gene clusters.

To identify coexpressed gene clusters on the genome, a sliding window analysis was performed using CROC software (94). For the sliding window, a step size of one gene and a window size of 12 genes were applied, of which at least 6 genes must be either up- or downregulated for these to be considered for further analysis. Consecutively, on these windows a hypergeometric test was performed to test for a nonrandom distribution of the up- or downregulated genes, which were considered coexpressed gene clusters. The resulting *P* values were corrected using a Benjamini-Hochberg multiple testing correction method (88).

### Data availability.

RNA-seq reads from this study are deposited in the NCBI SRA archive under PRJNA351919 (SRP093589). Accession numbers for data sets are as follows: sclerotia of strain SAS405, SRS1810933; primordia Apo12, SRS1810937; stipes Apo34, SRS1810938; apothecial disks Apo56, SRS1810941; ascospores, SRS1812737.
