# Supplementary material for: Functional Analysis of Mating Type Genes and Transcriptome Analysis during Fruiting Body Development of Botrytis cinerea
Source: mBio. 2018 Feb 13;9(1):e01939-17. doi: 10.1128/mBio.01939-17 (PMC5821092; doi:10.1128/mBio.01939-17)

Supplementary Figure 1

Schematic representation of strategy for targeted deletion of *MAT* genes.

Knockout of *BcMAT1-1-1*, *BcMAT1-1-5*, *BcMAT1-2-1* and *BcMAT1-2-4* by targeted gene replacement. Organization of *BcMAT1-1-1*, *BcMAT1-1-5*, *BcMAT1-2-1* and *BcMAT1-2-4* locus before and after homologous recombination. Orentation of the target gene and *HPH* are indicated by white and grey arrows, respectively. Upstream and downstream flanks of target genes are shown with open boxes. Polymerase chain reaction (PCR) analysis of wild-type strain SAS56, SAS405 and knockout mutant strains. The genomic DNA of each strain was used to verify 5' and 3' homologous recombination and absence of targeted genes in corresponding knockout mutants, respectively.

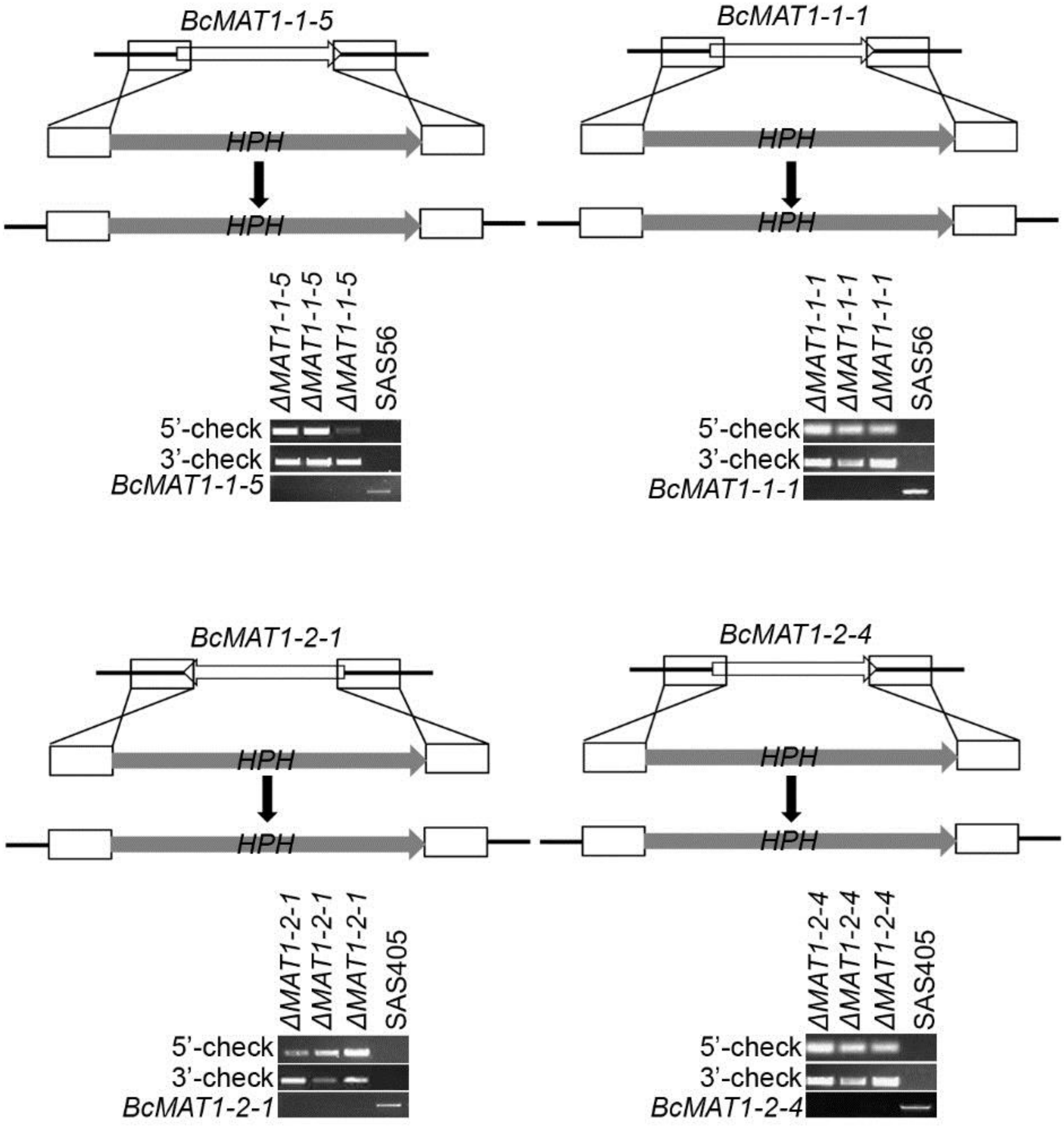

Supplement: FIG S1 [file mbo001183733sf1.pdf]
